# Positional Distribution of Fatty Acids in Triacylglycerols and Phospholipids from Fillets of Atlantic Salmon (*Salmo Salar*) Fed Vegetable and Fish Oil Blends

**DOI:** 10.3390/md13074255

**Published:** 2015-07-10

**Authors:** Noemi Ruiz-Lopez, Ingunn Stubhaug, Ignacio Ipharraguerre, Gerald Rimbach, David Menoyo

**Affiliations:** 1 Department of Agricultural Production, School of Agricultural Engineering, Technical University of Madrid, 28040 Madrid, Spain; E-Mail: noemi.ruiz@ig.csic.es; 2 Skretting Aquaculture Research Centre (ARC), P.O. Box 48, N-4001 Stavanger, Norway; E-Mail: Ingunn.Stubhaug@skretting.com; 3 Lucta S.A., Can Parellada 28, 08170, Montornés del Vallés, Barcelona, Spain; E-Mail: Ignacio.Ipharraguerre@lucta.es; 4 Institute of Human Nutrition and Food Science, University of Kiel, Hermann-Rodewald-Straße 6-8, D-24118 Kiel, Germany; E-Mail: Rimbach@foodsci.uni-kiel.de

**Keywords:** salmon (*Salmo salar*), omega 3 fatty acids, positional distribution, functional foods

## Abstract

The nutritional and functional characteristics of dietary fat are related to the fatty acid (FA) composition and its positional distribution in the triacylglycerol (TAG) fraction. Atlantic salmon is an important source of healthy long chain omega 3 FA (particularly, eicosapentaenoic (EPA) and docoxahexaenoic (DHA) acids). However, the impact of lipid sources in salmon feeds on the regiospecificity of FA in the fish TAG remains to be explored. The present study determines the effect of feeding salmon with blends of palm, rapeseed, and fish oil, providing two different EPA + DHA concentrations (high: H-ED 10.3% and low: L-ED 4.6%) on the fillet lipid class composition and the positional distribution of FA in TAG and phospholipids. The regiospecific analysis of fillet TAG showed that around 50% of the EPA and around 80% of DHA was located in the *sn*-2 position. The positional distribution of FA in phosphatidylcholine (PC), showed that around 80% of the EPA and around 90% of DHA were located in the *sn*-2. Fish fed the vegetable-rich diets showed higher EPA in the *sn*-2 position in PC (77% *vs*. 83% in the H-ED and L-ED diets, respectively) but similar DHA concentrations. It is concluded that feeding salmon with different EPA + DHA concentrations does not affect their positional distribution in the fillet TAG.

## 1. Introduction

Regiospecificity refers to the positioning of a single fatty acid in the glycerol backbone to conform triacylglycerols (TAG) and phospholipids (PL) [1]. In human nutrition there is increasing interest in studying the regiospecificity of fatty acids in foods as it plays an important role in their function and availability [2,3]. Fish oil (FO) is a rich source of long chain omega 3 fatty acids with proven beneficial cardiovascular effects [4,5]. Both eicosapentaenoic acid (EPA; C20:5*n*-3) and docosahexaenoic acid (DHA; C22:6*n*-3) are key fatty acids for the functional effects of FO [6]. Recently it has been shown that the lipid-lowering effects of FO are related to the positional distribution of EPA and DHA in TAG [7]. Moreover, it seems that DHA bound in the *sn*-2 position of the TAG can decrease serum cholesterol and TAG levels in mice [7]. Oily fish such as Atlantic salmon are an important source of EPA and DHA for consumers [8]. The dietary fatty acid profile is the main factor determining fatty acid composition in farmed salmon [8,9]. Therefore, EPA and DHA concentrations in salmon tissues are proportional to the amount of dietary FO [8,9]. However, because of market pressure on this raw material and sustainability issues, inclusion levels of FO in commercial salmon feeds have decreased in favor of vegetable oils (VO) [10]. Although VO-based diets have been shown to have no detrimental effect on salmon growth or health, their use results in decreased flesh EPA + DHA concentrations, lowering the nutritional quality of the fillet [9,10,11]. However, to our knowledge, there is a lack of studies in salmon assessing the effects of dietary oil sources on the positional distribution of fatty acids in TAG and PL. 

The positional distribution of fatty acids in TAG can be analyzed by enzymatic hydrolysis, chemical, Nuclear Magnetic Resonance (NMR) spectroscopic, and spectrometric methods [12]. Both enzymatic methods with lipases and ^13^C NMR have been used for FO TAG analysis [13,14]. However, the enzymatic method with pancreatic lipase may give rather unreliable results in FO with high DHA (30%) concentrations because of incomplete hydrolysis [14]. Both methods offer some advantages and disadvantages, but enzymatic methods are widely used as they are relatively simple, inexpensive, and do not need any special equipment [12]. The present study aims to determine the impact of feeding salmon with blends of palm, rapeseed, and FO providing two EPA + DHA concentrations (high EPA + DHA: H-ED 10.3% and low EPA + DHA: L-ED 4.6%) on the positional distribution of fatty acids in TAG and PL. In addition, the two methods for FO analysis, enzymatic with pancreatic lipase and ^13^C NMR, are compared.

## 2. Results and Discussion 

Fish performance was good and not affected by dietary treatments. Overall the specific growth rate and feed conversion ratio were 1.55 and 0.83, respectively. A lack of effect of dietary VO in farmed salmon productive performance has been extensively reported, as reviewed in [11].

### 2.1. Lipid Content and Fatty Acid Compositions of Fillets 

No effect of dietary oil was observed on the fillet’s total fat content (Table 1). As expected, the fatty acid composition of the salmon fillet reflected that of the diet, with significantly higher concentration of the characteristic fatty acids from FO, C14:0, EPA, C22:1 and DHA in fish fed the H-ED diet (Table 1). It is well known that fatty acid composition is influenced by dietary oil source; therefore, our results are in agreement with previous studies [8,9,11]. As a result, fish fed the H-ED diet accumulated about 8.1% of DHA and 3.1% of EPA of total fatty acids; however, fish fed L-ED accumulated about 4.9% and 1.5% of these fatty acids, respectively.

Fillet lipid contents have been shown to decrease in salmon fed with VO depending on the specific oil source or VO blend used [15], the genetic background of the fish (*i.e.*, lean or fat) [16], and the level of plant protein included in the diet [17]. In the present study, diets contained low fish meal (10% in final feed formulations) and FO levels (around 12% and 2% of total added oil in the H-ED and L-ED diets, respectively) blended with palm and rapeseed oils. These two vegetable oils have been shown to decrease fillet lipid contents when replacing 50% of the added FO [18,19]. In the present study both vegetable oils replaced around 70% of the FO compared with the H-ED diet, with no effects on flesh adiposity or lipid class composition.

**Table 1:** Fatty acid compositions (percentage of total fatty acids) of total lipids and lipid content (percentage of fresh weight FW) of fillets of salmon fed diets with high (H-ED) and low (L-ED) levels of EPA and DHA fatty acids.

	H-ED	L-ED	*p*-Value ^a^
C14:0	3.8 ± 0.1	1.9 ± 0.1	0.012
C16:0	14.8 ± 0.6	15.2 ± 0.6	0.685
C16:1*n*-7	3.3 ± 0.1	1.8 ± 0.1	0.021
C18:0	3.4 ± 0.1	3.5 ± 0.1	0.581
C18:1*n*-9	35.7 ± 0.5	44.4 ± 0.4	0.061
C18:1*n*-7	2.2 ± 0.3	1.7 ± 0.1	0.056
C18:2*n*-6	11.4 ± 0.4	13.3 ± 0.4	0.095
C18:3*n*-3	3.4 ± 0.1	3.3 ± 0.1	0.732
C18:4*n*-3	1.0 ± 0.1	0.8 ± 0.1	0.127
C20:1*n*-9	6.9 ± 0.2	4.5 ± 0.2	0.021
C20:2*n*-6	1.0 ± 0.0	1.0 ± 0.0	0.283
C20:3*n*-6	0.4 ± 0.0	0.8 ± 0.0	0.013
C20:4*n*-6	0.3 ± 0.0	0.3 ± 0.0	0.681
C20:3*n*-3	0.3 ± 0.0	0.2 ± 0.0	0.105
C20:4*n*-3	0.9 ± 0.0	0.5 ± 0.0	0.019
C20:5*n*-3	3.1 ± 0.1	1.5 ± 0.1	0.018
C22:1	7.6 ± 0.3	3.3 ± 0.3	0.010
C22:5*n*-3	1.3 ± 0.1	0.6 ± 0.1	0.024
C22:6*n*-3	8.1 ± 0.1	4.9 ± 0.1	0.005
∑SFA ^b^	22.5 ± 0.9	21.1 ± 0.9	0.398
∑MUFA ^c^	56.1 ± 2.3	56.2 ± 2.3	0.989
∑*n*-3 ^d^	18.3 ± 0.4	12.1 ± 0.4	0.009
∑*n*-6 ^e^	13.5 ± 0.5	16.1 ± 0.5	0.070
*n*3/*n*6	1.4 ± 0.01	0.8 ± 0.01	0.001
%TL (FW)	11.0 ± 0.4	10.5 ± 0.4	0.469

^a^ (mean ± SE; 8 fillets per diet; *n* = 2 tanks per diet); ^b^ ∑SFA = sum of saturated fatty acids. Includes C14:0, C16:0, C18:0 and C20:0; ^c^ ∑MUFA = sum of monounsaturated fatty acids. Includes C16:1*n*-9, C16:1*n*-7, C18:1*n*-9, C18:1*n*-7, C20:1*n*-9 and C22:1 isomers; ^d^ ∑ (*n*-3) = sum of *n*-3 fatty acids. Includes C18:3, C18:4, C20:3, C20:4, C20:5, C22:5 and C22:6; ^e^ ∑ (*n*-6) = sum of *n*-6 fatty acids. Includes C18:2, C20:2, C20:3 and C20:4.

### 2.2. Fillet Lipid Class Composition, TAG, and PC Fatty Acid Analysis 

The influence of these two experimental diets on lipid classes was also assessed in salmon fillets. However, no significant difference in lipid class composition was noted (Table 2). Triacylglycerol was identified as the predominant lipid class (about 92% in fillets of fish fed both experimental diets), followed by phosphatidylcholine (PC) (3.6% in fillets of fish fed both experimental diets). 

**Table 2:** Lipid class composition (% of total lipid) in the fillet of fish fed diets with high (H-ED) and low (L-ED) levels of EPA and DHA fatty acids ^a^*.*

	H-ED	L-ED	*p*-Value ^b^
TAG	92.3 ± 1.0	92.5 ± 1.0	0.864
DG	0.8 ± 0.1	0.7 ± 0.1	0.786
FFA	0.9 ± 0.2	1.0 ± 0.2	0.653
PC	3.7 ± 0.3	3.7 ± 0.3	0.927
PE	1.7 ± 0.1	1.6 ± 0.1	0.943
PI	0.3 ± 0.0	0.3 ± 0.0	0.642
PS	0.3 ± 0.0	0.2 ± 0.0	0.682

^a^ TAG, Triacylglycerol; DG, diacylglycerol; FFA, free fatty acids; PC, phosphatidylcholine; PE, phosphatidylethanolamine; PI, phosphatidylinositol; PS, phosphatidylserine; ^b^ (mean ± SE; 6 fillets per diet; *n* = 2 tanks per diet).

Additionally, the fatty acid composition of the TAG and PC fractions was also studied. The fatty acid composition of salmon fillet TAG reflected that of the diet, with significantly higher concentration of the characteristic fatty acids from FO, C14:0, EPA, C22:1 and DHA in fish fed the H-ED diet (Table 3). As mentioned above, it is well known that TAG composition is influenced by dietary oil source and especially in the fillet, given the high proportion of this lipid class (around 90%); therefore, our results are in agreement with previous studies [11,20]. 

**Table 3:** Fatty acid composition (% of total lipids) in the fillet TAG and PC of fish fed diets with high (H-ED) and low (L-ED) levels of EPA and DHA fatty acids ^a^*.*

	TAG			PC		
	H-ED	L-ED	*p*-Value ^a^	H-ED	L-ED	*p*-Value ^a^
C14:0	3.7 ± 0.0	1.9 ± 0.0	0.0003	2.2 ± 0.1	1.0 ± 0.1	0.003
C16:0	13.1 ± 0.3	14.0 ± 0.2	0.148	20.8 ± 0.2	22.5 ± 0.2	0.022
C16:1*n*-7	3.2 ± 0.1	2.0 ± 0.0	0.003	1.4 ± 0.1	0.7 ± 0.1	0.018
C18:0	2.7 ± 0.1	3.2 ± 0.1	0.053	0.9 ± 0.1	1.0 ± 0.1	0.581
C18:1*n*-9	32.5 ± 0.6	42.1 ± 0.5	0.005	11.7 ± 0.1	15.4 ± 0.1	0.010
C18:2*n*-6	10.9 ± 0.2	12.9 ± 0.2	0.015	3.8 ± 0.2	7.2 ± 0.2	0.008
C18:3*n*-3	3.2 ± 0.0	3.3 ± 0.0	0.024	2.0 ± 0.1	2.7 ± 0.1	0.025
C20:1*n*-9	6.4 ± 0.1	4.3 ± 0.1	0.001	0.8 ± 0.0	0.5 ± 0.0	0.031
C20:2*n*-6	0.8 ± 0.0	0.9 ± 0.0	0.365	0.5 ± 0.0	0.6 ± 0.0	0.095
C20:3*n*-6	0.3 ± 0.0	0.6 ± 0.0	0.016	0.6 ± 0.1	2.3 ± 0.1	0.004
C20:4*n*-6	0.2 ± 0.0	0.2 ± 0.0	0.249	0.7 ± 0.0	1.3 ± 0.0	0.005
C20:4*n*-3	0.8 ± 0.0	0.5 ± 0.0	0.008	0.9 ± 0.0	0.9 ± 0.0	0.935
C20:5*n*-3	2.5 ± 0.1	1.2 ± 0.1	0.008	10.8 ± 0.4	8.1 ± 0.3	0.031
C22:1	7.1 ± 0.1	3.3 ± 0.1	0.0009	0.3 ± 0.0	0.3 ±0.0	0.770
C22:5*n*-3	1.0 ± 0.0	0.5 ± 0.0	0.018	2.4 ± 0.2	2.1 ± 0.2	0.295
C22:6*n*-3	5.1 ± 0.2	2.9 ± 0.2	0.010	34.5 ± 01.0	28.0 ± 0.9	0.040
∑SFA ^b^	19.9 ± 0.3	19.6 ± 0.2	0.474	24.0 ± 0.2	24.7 ± 0.2	0.168
∑MUFA ^c^	52.1 ± 0.4	54.6 ± 0.4	0.037	15.8 ± 0.3	18.3 ± 0.3	0.034
∑*n*-3 ^d^	14.4 ± 0.2	9.6 ± 0.2	0.001	51.9 ± 0.5	44.4 ± 0.5	0.010
∑*n*-6 ^e^	12.2 ± 0.1	14.8 ± 0.2	0.002	5.9 ± 0.31	11.7 ± 0.28	0.005
*n*3/*n*6	1.2 ± 0.0	0.65 ± 0.0	0.001	8.7 ± 0.25	3.8 ± 0.23	0.004

^a^ (mean ± SE; 6 fillets per diet; *n* = 2 tanks per diet); ^b^ ∑SFA = sum of saturated fatty acids. Includes C14:0, C16:0, C18:0 and C20:0; ^c^ ∑MUFA = sum of monounsaturated fatty acids. Includes C16:1*n*-9, C16:1*n*-7, C18:1*n*-9, C18:1*n*-7, C20:1*n*-9 and C22:1 isomers; ^d^ ∑ (*n*-3) = sum of *n*-3 fatty acids. Includes C18:3, C18:4, C20:3, C20:4, C20:5, C22:5 and C22:6; ^e^ ∑ (*n*-6) = sum of *n*-6 fatty acids. Includes C18:2, C20:2, C20:3 and C20:4.

As described in the literature C16:0, C18:1*n*-9, EPA, and DHA were the principal fatty acids on PC [21] (Table 3). Although TAG is the lipid fraction more influenced by diet, the PL fatty acid profile is also affected by dietary oil composition [20,22]. Moreover, PC seems to be the PL most influenced by diet [21]. The concentration of C16:0 and C18:1*n*-9 was higher in the PC of fish fed the rich VO diet, while EPA and DHA values were higher in those fed the higher FO diet. Also, a significant increase of linoleic acid (C18:2*n*-6; LA) and their elongation products C20:3*n*-6, Arachidonic acid (C20:4*n*-6; AA) was noticeable in the PC fraction of fish fed the VO rich diet. This is in accordance with previous studies in Atlantic salmon fed diets including VO and FO [22]. 

### 2.3. Regiospecific Analysis of Salmon Fillet Triacylclycerol and Phosphatidylcholine Classes 

**Figure 1:**
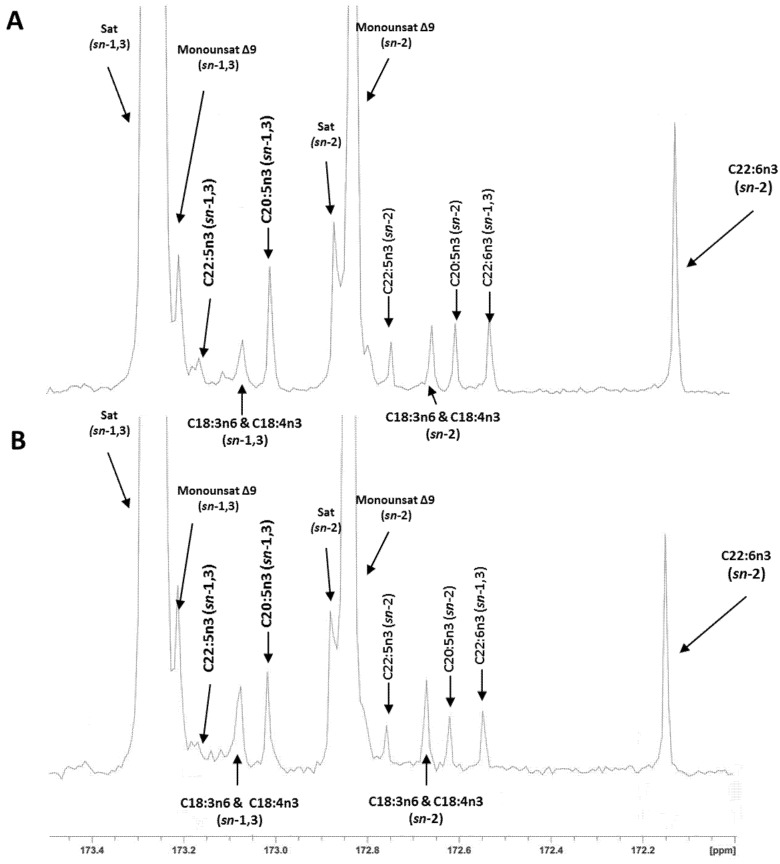
176.07 MHz ^13^C NMR spectra of the carbonyl region of (**A**) H-ED fillets and (**B**) L-ED fillet oils with the assigned *sn*-1,3 and *sn*-2 regioisomeric peaks to individual fatty acids.

The regiospecific analysis of fillet TAG showed that half of the EPA (49%–50%) and most of the DHA (80%–86%) was located in the *sn*-2 position of the TAG (Table 4). Reported values in the literature for these fatty acids in the *sn*-2 position of the TAG range from 34% to 47% for EPA and from 62% to 76% for DHA [1,13], which are slightly lower than our results, especially for DHA. In the present study, the analysis with lipase and ^13^C NMR method gave a similar EPA percentage in the *sn*-2 position of the TAG. However, a significant method effect (*p* < 0.002) was observed for DHA. A lower DHA percentage in the *sn*-2 position was reported with the pancreatic lipase than with the ^13^C NMR method: 81.7% *vs.* 86.3%, respectively. As previously indicated, values in the literature for DHA in the *sn*-2 position of salmon TAG analyzed by ^13^C NMR are up to 76% [1,13]. This value is similar to the one obtained in the present study with the lipase method (81.7%). However, it differs slightly from the results we obtained with the ^13^C NMR method (86.3%). Therefore, we cannot exclude some artifacts overestimating the values for DHA in our ^13^C NMR data, especially in the low DHA samples (*p* = 0.07 for the interaction). Assignment of carbon resonances and representative ^13^C NMR spectra of the carbonyl region of H-ED and L-ED salmon oils are presented in Figure 1A,B; a relaxation time of 10 s was chosen for comparison of carbons in the same electronic environment (carbonyl region). A recent study with hoki oil (*Macruronus novaezelandiae*) containing 10%–12% of DHA and 5%–6% EPA showed the same positional distribution of both fatty acids using pancreatic lipase and ^13^C NMR methods [14]. Thus with our study we can confirm pancreatic lipase as a reliable method for the analysis of fish oils with low DHA (<10%) concentration. 

**Table 4:** Proportion of EPA and DHA (molar %) in *sn*-2 position in the fillet TAG of fish fed diets with high (H-ED) and low (L-ED) levels of EPA and DHA fatty acids determined by pancreatic lipase or by ^13^C NMR spectroscopy.

	EPA	DHA
*Diet*		
H-ED	50.1 ± 1.3	84.9 ± 0.7
L-ED	49.3 ± 1.3	83.1 ± 0.7
Method		
Lipase	50.1 ± 1.1	81.7 ± 0.7
NMR	49.2 ± 1.0	86.3 ± 0.6
*Diet × Method*		
H-ED Lipase	50.7 ± 1.6	83.5 ± 1.0
H-ED NMR	49.4 ± 1.4	86.3 ± 0.8
L-ED Lipase	49.4 ± 1.4	79.9 ± 1.0
L-ED NMR	49.1 ± 1.4	86.4 ± 0.8
*Probability* ^a^		
Diet	0.7286	0.1377
Method	0.4323	0.0020
Diet × Method	0.6219	0.0758

^a^ mean ± SE; 4 fillets per diet; *n* = 2 tanks per diet.

No significant changes on the proportions of EPA and DHA in the *sn*-2 position of the fillet TAG were observed with the experimental diets fed to salmon. Among rearing conditions, water temperature seems to have a direct effect on DHA positional distribution with higher temperatures (*i.e.*, 19 °C), decreasing the percentage of this fatty acid in the *sn*-2 position [1]. Results from this study suggest that feeding Atlantic salmon blends of FO and VO providing high or low EPA and DHA have no effect in the positioning of both fatty acids in the TAG. 

Additionally, the positional distribution of fatty acids in PC showed that most of the EPA (around 80%) and DHA (around 90%) were located in the *sn*-2 position of the PC (Table 5). Saturated fatty acids C14:0 and C16:0 were predominantly in the *sn*-1 position given the low levels found in the *sn*-2 position in the PC. This is in accordance with previous reports in salmon where the major molecular species in muscle PC is 16:0/22:6 [1]. However, it also agrees with the important role that EPA and especially DHA play as structural lipids in fish membranes, as they provide the sufficient plasticity to cope with pressure and temperature changes in the water environment [21]. Dietary effects were evident on fatty acid positional distribution in the PC. Fish fed the low EPA and DHA diets with higher dietary concentrations of LA and linolenic (C18:*3n-3*) acids tended to show a higher proportion of both fatty acids in the *sn*-2 position compared to those fed the high EPA and DHA diet rich in FO. Also, higher concentrations of Arachidonic acid (AA) in the PC of fish fed the VO-rich diet tended (*p* = 0.08) to accumulate in the *sn*-2 position. Increasing levels of AA can lead to undesired side effects on tissue adiposity and the fish immune and antioxidant status [22,23]. Phospholipases hydrolyze the ester bond at the *sn*-2 position of the glycerophospholipid releasing free AA for eicosanoid production [24]. Furthermore, it has been shown that salmon fed on a sunflower oil diet had increased phospholipase A activity [22]. Therefore, future research is needed to study the possible implications of AA positioning in salmon membranes on the immune and antioxidant response.

**Table 5:** Proportion of selected fatty acids (FAME%) in *sn*-2 position in the fillet PC of fish fed diets with high (H-ED) and low (L-ED) levels of EPA and DHA fatty acids.

	H-ED	L-ED	*p*-Value ^a^
C14:0	21.3 ± 2.6	19.5 ± 2.6	0.680
C16:0	16.4 ± 1.9	13.3 ± 1.9	0.378
C18:1*n*-9	52.6 ± 1.4	57.4 ± 1.4	0.150
C18:2*n*-6	45.5 ± 2.3	58.1 ± 2.3	0.063
C18:3*n*-3	25.7 ± 1.0	31.2 ± 1.0	0.063
C20:3*n*-6	53.5 ± 1.3	66.3 ± 1.3	0.022
C20:4*n*-6	89.1 ± 0.8	93.2 ± 0.8	0.080
C20:5*n*-3	77.4 ± 0.9	83.5 ± 0.9	0.040
C22:5*n*-3	80.3 ± 1.3	85.7 ± 1.3	0.099
C22:6*n*-3	89.7 ± 0.9	91.6 ± 0.9	0.312

^a^ mean ± SE; 4 fillets per diet; *n* = 2 tanks per diet.

Fish fed the VO rich diet showed higher EPA concentration in the *sn*-2 position (77% *vs.* 83% in H-ED and L-ED, respectively) and similar (89% *vs.* 91%) DHA concentrations. Thus, it seems that when the dietary input of EPA and DHA is lower, salmon tend to retain both fatty acids in the *sn*-2 position of the phospholipid given the lower availability and the important structural role for both fatty acids, as previously indicated.

## 3. Experimental Section 

### 3.1. Animals and Diets 

The feeding trial was run at Skretting Aquaculture Research Center (ARC) fish trial station (Lerang Research Station, Jorpeland, Norway). A total of 128 Atlantic salmon post-smolts with an average initial weight of 148 ± 3 g were randomly assigned to two experimental diets. All experimental procedures were performed according to the Norwegian Animal Research Authority (FDU) guidelines. Fish were randomly assigned to four 1 m deep × 1 m diameter circular tanks supplied with sea water and fed one of the two experimental diets for 15 weeks. Water temperature over the experimental period averaged 11.8 ± 0.3 °C. Diets were formulated to meet salmon nutrient recommendations [25] by using low fishmeal and blends of palm, rapeseed, and fish oil providing two EPA + DHA concentrations (high: H-ED 10.3% and low: L-ED 4.6%) (Table 6). The dietary treatments were prepared at Skretting ARC pilot plant (Stavanger, Norway), as extruded, sinking 4 mm pellets.

**Table 6:** Ingredients and analyzed chemical composition of the basal diet.

Ingredients (as Fed Basis g/kg)	H-ED	L-ED
Wheat ^a^	35.0	35.0
Wheat gluten ^b^	149.6	149.9
Sunflower meal ^c^	88.4	84.8
Fava beans, dehulled ^d^	60.0	60.0
Soy Protein Concentrate ^e^	310.0	310.0
Fishmeal NA ^f^	100.0	100.0
Palm oil ^g^	2.1	46.7
Rapeseed oil ^h^	91.9	146.5
Fish oil NA ^i^	123.1	26.1
Astaxanthin 10% ^j^	0.4	0.4
Vitamin and mineral mix ^k^	32.1	32.1
Water	7.1	7.4
*Analyzed composition*		
Moisture, %	6.4	6.6
Total fat, %	25.1	25.5
Crude Protein, %	45.2	45.2
Ash, %	4.5	4.6
*Fatty acids (g/100 g total fatty acids)*		
C14:0	3.8	1.7
C16:0	10.6	13.0
C18:1*n*-9	27.3	38.8
C18:2*n*-6	11.8	16.3
C18:3*n*-3	4.2	5.2
C20:5*n*-3	4.8	2.1
C22:6*n*-3	5.5	2.5
∑SFA ^l^	17.7	17.8
∑MUFA ^m^	47.2	49.8
∑ (*n*-3)	17.2	10.8
∑ (*n*-6)	12.8	16.7
*n*-3/*n*-6	1.34	0.64

^a^ HaGe Kiel AG, Germany; ^b^ Cargill Cerestar, Hautbourdin, France; ^c^ Rosenkrantz AS, Aalborg, Denmark; ^d^ Soufflet, Nogent-sur-Seine, France; ^e^ Imcopa-Imp.Exp.e, Araucaria PR, Brazil; ^f^ Scandinavian fish meal, Welcon, Norway; ^g^ AAK Karlsham, Karlsham, Sweeden; ^h^ Linas Agro AS, Vilnius, Lithuania; ^i^ Northern hemisphere fish oil, Måløy Sildeoljefab, Måløy, Norway; ^j^ DSM, Heerlen, The Netherlands; ^k^ Include vitamins and minerals; Trouw Nutrition, Boxmeer, The Netherlands, proprietary composition Skretting ARC, vitamin and mineral supplementation as estimated to cover requirements according NRC (2011); ^l^ ∑SFA = sum of saturated fatty acids; ^m^ ∑MUFA = sum of monounsaturated fatty acids.

### 3.2. Sampling

At the beginning and end of the experimental period, all fish were individually weighed and measured for growth monitoring. Fish were deprived for feed for 12 h after the final sampling and then anesthetized with Tricaine Pharmaq (Pharmaq Ltd., Hampshire, UK) and killed by a blow to the head. Four fish per tank (*n* = 8 per diet) were selected to obtain individual samples of fillet. Tissues were collected, snap frozen in dry ice, and stored at −80 °C for fatty acid analysis. 

### 3.3. Feed Analysis 

Feed proximal analysis including moisture, total fat, protein, and ash was performed by using in-house near-infrared reflectance (NIR) methodology at the Skretting ARC laboratory, as previously described by Torstensen *et al*. [26]. Fatty acid analysis of the experimental diets was conducted using gas chromatography and flame ionization detection [27]. 

### 3.4. Tissue Total Lipid Extraction and Analysis

Total lipids were extracted from fillet samples by the method of Segura and López-Bote [28]. Lyophilised samples (200 mg) were homogenized in dichloromethane:methanol (8:2, by vol.) using a mixer mill (MM400, Retsch technology, Haan, Germany). The final biphasic system was separated by centrifugation. The extraction was repeated three times. Solvent was evaporated under nitrogen stream and lipids were dried by vacuum desiccation. The total lipid content was determined gravimetrically. 

Fatty acid methyl esters (FAME) were prepared from total lipids by transesterification using a mixture of sodium methylate:methanol and heptadecanoic acid (17:0) as an internal standard. Samples were heated at 70 °C for 1 h. Samples were re-heated for 1 h at 70 °C after adding 5% sulfuric acid in methanol. FAMES were extracted with petroleum ether and separated by GC-FID.

### 3.5. Lipid Classes Analysis

Major lipid classes from total lipid samples were separated by one-dimensional thin-layer chromatography (TLC), as previously described by Henderson and Tocher [29] with minor modifications. Total lipid samples (2–3 mg) were applied to TLC silica plates with fluorescent indicator (20 cm × 20 cm × 250 µm, Macherey-Nagel, Düren, Germany). Plates were developed to 9.5 cm in methyl acetate:isopropanol:chloroform:methanol:0.25% aqueous KCl (25:25:25:10:9, by volume). Excess solvent was evaporated via air drying and plates were further developed using a solvent mixture containing hexane: diethyl ether: acetic acid (80:20:1, by volume). The individual lipid classes were identified under UV light after a Primuline spray by comparison with commercial standards, as previously described [30]. Lipid fractions were scraped from the plate and used directly for methylation or extracted for further analysis. FAME compositions of these isolated lipid fractions were obtained by heating the samples at 80 °C for 1 h in methanol:toluene:H_2_SO_4_ (88:10:2, by vol.) as in [31]. After cooling, FAME were recovered with hexane and separated and quantified by GC-FID.

### 3.6. Positional Analysis of TAG

#### 3.6.1. ^13^C NMR Spectroscopy

Quantitative broad band ^13^C NMR spectra from previously purified TAG were recorded under continuous 1H decoupling at 25 °C in a Bruker AV-III 700 MHz spectrometer, equipped with a 5 mm TCI cryoprobe using the standard TopSpin V 2.1 software, as previously described by Suarez *et al*. [32] with minor modifications. The spectrometer was located at the Nuclear Magnetic Resonance Centre (one of the Complutense University of Madrid Research Support Centers, Madrid, Spain). The data were acquired at a ^13^C frequency of 176.07 MHz using the following acquisition parameters: 128 k complex data points, spectral width of 41,666 Hz (237 ppm), pulse width 90°, acquisition time 1.573 s, and collection of 2048 scans. A repetition time of 10 s was employed. Prior to Fourier transformation, all free induction decays (FID) were zero filled to 128 k real data points and apodized using exponential multiplication (0.3–0.7 Hz line broadening) for sensitivity enhancement.

#### 3.6.2. Enzymatic Analysis

Positional analysis of purified TAG was performed as described by Luddy *et al*. [33] and adapted by Ruiz-Lopez *et al*. [34]. Samples containing 5 mg TAG were dried under nitrogen and resuspended in 1mL of 1 mM Tris-HCl (pH 8.0). Samples were then sonicated for 60 s to ensure complete emulsification of the lipid. Then, 0.1 mL of 22% CaCl_2_ and 0.25 mL of 0.1% deoxycholate were added. Samples were warmed at 40 °C for 30 s, and 2 mg pancreatic lipase (≥20,000 units per mg protein; Sigma, St. Louis, MO, USA) was added. Samples were vortexed for 2–3 min. The reaction was terminated using 0.5 mL 6 M HCl. The lipids were extracted twice with 2.5 mL diethyl ether. Lipids were evaporated at 40 °C under nitrogen and separated into lipid classes by TLC using silica plates and hexane/diethyl ether/acetic acid (70:30:1 by vol.). The spots corresponding to 2-monoacylglycerols were identified under UV light after a Primuline spray, scraped from the plate, and directly transmethylated for GC-FID analysis. The mean composition of fatty acids in the *sn*-1,3 positions was calculated using the composition of an aliquot of the initial triacylglycerol and the formula: mean percentage *sn*-1,3 = [(3 × % fatty acid in triacylglycerol) − (% fatty acid in the *sn*-2 position)]/2. The validity of the procedure was confirmed by comparing the FA composition of the intact TAG sample and those remaining after the partial hydrolysis [35].

### 3.7. Positional Analysis of PC

Positional analysis of purified PC was performed as described previously by Ruiz-Lopez *et al*. [36]. Briefly, analysis of PC was performed using *Naja mossambica* phospholipase A2 (Sigma, St. Louis, MO, USA). Samples containing PC were dried under nitrogen and resuspended in 1 mL borate buffer (0.5 M, pH 7.5, containing 0.4 mM CaCl_2_) by sonication. Five units of lipase and 2 mL diethyl ether were added, and the digestions were performed by vortexing for 2 h. The ether phase was evaporated, and the reaction was stopped by adding 0.3 mL of 1 M HCl. The aqueous phase was extracted using chloroform:methanol (2:1, by volume). The resulting organic phase was dried under nitrogen and separated by TLC using chloroform:metanol:aqueous ammonia (65:25:0.7, by volume) as the solvent mix. The spots corresponding to free fatty acids and lysophospholipids were scraped from the plate and directly transmethylated for GC-FID analysis (as described above).

### 3.8. Fatty Acid Methyl Esters Analysis

FAMEs were separated using a gas chromatograph (HP 6890 Series GC System) equipped with flame ionization detector. Separation was performed with a J&W GC Column, HP-Innowax Polyethylene Glycol (30 m × 0.316 mm × 0.25 μm). Nitrogen was used as a carrier gas. After injection at 170 °C, the oven temperature was raised to 210 °C at a rate 3.5 °C/min, then to 250 °C at a rate of 7 °C/min and held constant for 1 min. The flame ionization was held at 250 °C. FAME peaks were identified by comparing their retention times with those of authentic standards (Sigma-Aldrich, Alcobendas, Spain).

### 3.9. Statistical Analysis

Differences between means were detected through a mixed-model procedure of SAS (release 9.2, SAS Institute Inc., Cary, NC, USA). For fatty acid analysis the model included the diet as fixed effect and the tank was considered a random effect. To compare pancreatic lipase and ^13^C NMR the model included diet and method as fixed effects and the tank was considered a random effect. 

## 4. Conclusions 

Blends of FO and VO providing different concentrations of EPA + DHA (10.3% *vs.* 4.6%) fed to Atlantic salmon have no effect on the proportion of both fatty acids in the *sn*-2 position of fillet TAG. However, dietary effects were evident on fatty acid positional distribution in the PC. In fish fed the blend rich in VO the AA tended to accumulate in the *sn*-2 position of PC. Also, when dietary input of EPA and DHA is lower, salmon significantly retain both fatty acids in the *sn*-2 position of the PC given the lower availability and the important structural and metabolic role of this position for both fatty acids.
